# Factors influencing use of long-acting versus short-acting contraceptive methods among reproductive-age women in a resource-limited setting

**DOI:** 10.1186/s12905-017-0382-2

**Published:** 2017-04-04

**Authors:** Leevan Tibaijuka, Robert Odongo, Emma Welikhe, Wilber Mukisa, Lilian Kugonza, Imelda Busingye, Phelomena Nabukalu, Joseph Ngonzi, Stephen B. Asiimwe, Francis Bajunirwe

**Affiliations:** 1 Department of Obstetrics and Gynecology, Mbarara University of Science and Technology, Mbarara, Uganda; 2 Department of Internal Medicine, Mbarara Regional Referral Hospital, Mbarara, Uganda; 3 Department of Epidemiology and Biostatistics, University of California San Francisco, California, USA; 4 Mbarara University of Science and Technology, Mbarara, Uganda

**Keywords:** Long-acting reversible contraceptives (LARC), Short-acting contraceptives, Method selection, Factors, Resource-limited setting

## Abstract

**Background:**

Unplanned pregnancy remains a common problem in many resource-limited settings, mostly due to limited access to modern family planning (FP) services. In particular, use of the more effective long-acting reversible contraceptive (LARC) methods (i.e., intrauterine devices and hormonal implants) remains low compared to the short-acting methods (i.e., condoms, hormonal pills, injectable hormones, and spermicides). Among reproductive-age women attending FP and antenatal care clinics in Uganda, we assessed perceptions and practices regarding the use of modern contraceptive methods. We specifically aimed to evaluate factors influencing method selection.

**Methods:**

We performed a mixed-methods cross-sectional study, in which we administered structured interviews to 180 clients, and conducted 4 focus group discussions (FGDs) with 36 clients and 8 in-depth personal qualitative interviews with health service providers. We summarized quantitative data and performed latent content analysis on transcripts from the FGDs and qualitative interviews.

**Results:**

The prevalence of ever use for LARC methods was 23%. Method characteristics (e.g., client control) appeared to drive method selection more often than structural factors (such as method availability) or individual client characteristics (such as knowledge and perceptions). The most common reasons for choosing LARC methods were: longer protection; better child-spacing; and effectiveness. The most common reasons for not choosing LARC methods included requiring a client-controlled method and desiring to conceive in the near future. The most common reasons for choosing short-acting methods were ease of access; lower cost; privacy; perceived fewer side effects; and freedom to stop using a method without involving the health provider. The personal characteristics of clients, which appeared to be important were client knowledge and number of children. The structural factor which appeared to be important was method availability.

**Conclusions:**

Our results suggest that interventions to improve uptake of LARC among reproductive age women in this setting should consider: incorporating desired method-characteristics into LARC methods; targeted promotion and supply of LARC; and increased counselling, sensitization, and education.

## Background

Resource limited settings such as Uganda are currently experiencing rapid population growth mainly due to high fertility rates and limited access to contraception [1]. In Uganda, the annual population growth rate is 3.2% and the country has one of the highest total fertility rates (6.2 children per woman) in the world [2]. Concurrently, the use of modern contraceptive methods is low. For example, among sexually active women desiring contraception, only 31% use modern contraceptive methods; 61% lack access; the remainder use traditional methods [3].

Modern contraceptive methods can be classified under 3 broad groups: 1) long-acting reversible contraceptives (LARC), i.e., intrauterine devices (IUD) and hormonal implants; 2) short-acting contraceptives (i.e., oral contraceptive (OC) pills, condoms, spermicides, and injectable hormones; and 3) permanent methods (i.e., sterilization via tubal-ligation or vasectomy [1]. In all settings, short-acting methods are more commonly used than LARC methods, despite the LARC methods being more efficacious, more cost-effective, and better tolerated than short-acting methods [4–6]. Further, the effectiveness of short-acting methods is highly dependent on user characteristics such as education level [7, 8]. Consequently, short-acting methods can be less effective in resource-limited settings where many women seeking contraception may have low education levels [7]. In these settings, LARC methods should be encouraged, since the effectiveness of such methods is often independent of user characteristics [8].

While we know that use of these methods should be encouraged, we also know that their use in resource-limited settings remains low. In a previous study in Uganda, only 3.2% of respondents were using either an intrauterine device or a hormonal implant [3]. Although this could be due to lack of access to the methods [9], some previous studies suggest that women in resource-limited settings may simply prefer short-acting methods over LARC methods [3]. The factors driving such preference have not been clarified.

Among reproductive-age women attending family planning (FP) and antenatal care (ANC) clinics in the district of Mbarara, in South Western Uganda, we assessed perceptions and practices regarding the use of modern contraceptive methods. Our primary objective was to assess the factors influencing method selection. A better understanding of such factors could facilitate the design of future interventions to increase the uptake of LARC methods in this and other resource-limited settings. A secondary objective was to determine the prevalence of LARC methods use in a resource-limited setting.

## Methods

### Study design and setting

We conducted a mixed methods cross sectional study at 6 public health centers in Mbarara district, Uganda, involving quantitative interviews and focus group discussions with clients, and qualitative interviews with health workers. We purposively selected sites to represent rural and urban settings. In the urban setting, we selected 4 sites; the largest health center from each of the three divisions of Mbarara Municipality (Kakoba health center III from Kakoba, Nyamitanga health center III from Nyamitanga and Mbarara Municipal Council (MMC) health center IV from Kamukuzi), as well as Mbarara Regional Referral Hospital (MRRH), a tertiary referral hospital. The rural setting has two counties (Rwampara and Kashari); we selected the largest health center (health center IV) per county (Kinoni health center IV in Rwampara and Bwizibwera health center IV in Kashari). All participants provided written informed consent to participate in the study. The study was also approved by the institutional review committee of Mbarara University of Science and Technology.

### Study population

Participants included women aged 15 to 49 (client respondents), who were attending family planning and antenatal care clinics at the 6 participating health centers in June to August 2013, as well as the service providers at these clinics (8 service providers selected from across the participating health centers). Eligible client respondents were currently using or having ever used any modern contraceptive method. Those seeking services outside of the clinics’ working hours (8:00 am to 2:00 pm) were excluded. Eligible women were invited to participate; those consenting were consecutively enrolled. A separate purposive sample of 36 clients participated in 4 focus group discussions (6 to 12 participants per group).

#### Recruitment procedures

The interviews took place on clinic days. In the rural setting, there was one clinic day per week with an attendance of about 15 people per clinic day. Quantitative interviews at the rural centers were conducted by one interviewer per center, who interviewed 7–8 people per day completing the process over 4 clinic days. At the beginning of each clinic day, clients are usually brought together in a common area for a group counselling session. We approached women to participate in the interviews while they waited in the common area for this group counselling session. We then conducted the interviews either before or after the counseling session, depending on the client’s choice. The first 7–8 people that agreed to participate on each clinic day were interviewed. At the urban centers, the procedure was similar, except that antenatal care and family planning services at these centers are provided daily. The interviews at the 4 urban centers were conducted by 4 interviewers, with one interviewer stationed at each center. We conducted the interviews on 3 consecutive days aiming to interview up to 10 clients per day until the targeted sample of about 30 people per center was reached.

### Measurements

#### Quantitative measurements

Client respondents completed an interviewer-administered structured questionnaire, which we developed and pre-tested prior to data collection. The questionnaire was designed to collect some social and demographic information about the clients and to assess their knowledge, attitudes, and practices regarding the use of contraceptive methods. Knowledge, attitudes, and practices were assessed using both open ended and closed ended questions. There were 6 questions assessing knowledge, all closed ended (e.g., from the following list of methods, which one(s) do you know?), and 4 questions assessing attitudes, all closed ended (e.g., state whether you agree or disagree with the statement “long-acting methods are more effective than short-acting methods”). A total of 13 questions assessed practices; 9 were closed ended (e.g., are you currently using a method of contraception?) and 4 were open ended (e.g., why did you chose to use a long-acting method?).

#### Assessing knowledge

We provided the clients with a list of contraceptive methods (oral pills; injectables; condoms; implants; vasectomy; tubal ligation; spermicides; intrauterine devices; and withdrawal method). We then asked the participants to state whether they knew how these methods are used. For this question, participants were given three options: “I know how all the methods are used”; “I know how some of the methods are used”, and “I do not know how any of the methods is used”. Next, we asked them to re-list the methods under two groups: short-term and long-term, depending on how long they thought the method’s effects would last.

#### Assessing attitudes

First, we asked participants whether or not they agreed or disagreed with 3 statements (“long-term methods are more effective than short-term methods”; “contraceptives cause birth defects”; and “contraceptives cause cancer”). Additional questions to assess attitudes asked participants to state whether they thought that long-term methods were more effective than short-term methods, to state what they thought was the most effective method from a list of options, and what they thought was the least effective method from the same list.

#### Assessing practices

Practices were assessed using questions on current method, previous method(s), duration of use for each, and reasons for choosing or changing methods.

All questions were translated to the local language (Runyankole) prior to data collection; quantitative responses were copied from the questionnaire directly into a computer program (Microsoft Excel, Redmond, Washington) in which corresponding variables had been developed.

#### Qualitative data collection

We performed a total of 4 focus group discussions (FGD) with a total of 36 clients (6–12 participants per FGD) and 8 in-depth personal interviews with health providers. Qualitative interviews were conducted by two investigators in a quiet environment; one asked questions as the other took notes. Example questions included “why do you think women are opting to use short-term methods more than long-term methods?”.

We conducted 2 FGDs at a rural site (Kinoni health center IV), and 2 at an urban site (Mbarara Municipal Council health center IV); at each site, one FGD was with ever-users of a LARC, while the other was with ever-users of a short-acting method. For the FGDs, we invited clients who had not participated in the quantitative interviews. For short-acting methods users, potential participants were many and were readily available; we thus recruited them in each clinic’s waiting area as already described for the quantitative interviews. LARC users were not many on any given clinic day; we thus used clinic registers to identify individuals using LARC methods. We then requested clinic charge nurses or midwifes to help us contact the participants and request for their participation in the FGDs. We then organized and conducted the FGDs on days that were convenient for all participants.

Each FGD was led by one researcher, while an assistant took notes. The FGD opened with general questions such as, “let us talk about the methods of contraception available in this setting” before delving into more specific questions such as “what do you think are the advantages and disadvantages of different methods?”, and “when you chose to use the method that you are using, can you tell us why you chose that method and not another?”.

### Data analysis

#### Quantitative data analysis

For the 180 client respondents who participated in quantitative interviews, we summarized numeric and categorical characteristics as frequencies and percentages. We also tabulated frequencies of all reasons given during structured interviews for choosing or not choosing each type of method (LARC versus short-acting). We performed bivariate analyses comparing proportions of LARC users in different categories of variables of interest such as more educated vs. less educated clients, those with more children vs. those with fewer children. These analyses were stratified by setting, i.e., urban vs. rural. We used chi-squared tests to assess for whether differences were statistically significant at *P* < 0.05. To assess for factors associated with use of LARC methods, we compared the odds of having ever used a LARC in different levels of selected characteristics using logistic regression. For example we compared the odds of having ever used a LARC among those with ≥4 children to the odds of having ever used a LARC among those with 0–1 child.

#### Missing data

Education level was not assessed at the rural health centers (the questionnaire was modified to include this variable after quantitative data had already been collected from these centers); this field remained missing in the final dataset for all clients from rural health centers. Other missing quantitative data were obtained from client files at the health centers. The data available in these records were age, religion, and address. All quantitative analyses were performed using Stata 13, College Station, Texas.

#### Qualitative data analysis

Interviews with health workers occurred in English, while focus group discussions with clients occurred in Runyankole. Both sets of data were audio-recorded and transcribed afterwards; the Runyankole transcripts were translated to English by a person fluent in both English and Runyankole. English transcripts and available notes were then analyzed manually for content using an emergent approach and latent content analysis [10]. The analyst read through each transcript several times highlighting and labelling blocks of text with related underlying meaning (codes). The identified codes were then subjected to constant comparison [11] before being merged into categories of codes with related meaning. The themes connecting the codes within each category were then identified and are reported descriptively.

## Results

### Quantitative data

#### Response rates

Between June and August 2013, we approached and invited a total of 222 clients to participate in quantitative interview and focus group discussions, and 8 health workers to participate in qualitative in-depth interviews. All of those invited to participate in quantitative interviews accepted with the exception of 2 urban clients, who declined due to lack of time. Also, all short-acting methods users approached agreed to participate in the FGDs. As long-acting methods users were recruited using phone-calls in both settings, some of those contacted ended up not showing up for the FGDs, leading to about 67.0% response rate in both settings on this activity.

#### Subject characteristics

The characteristics of 180 clients who participated in quantitative interviews at the 6 health centers are summarized in Table 1. Median age was 28 years (IQR 23–32) among the rural clients and 25 years (IQR 23–30) among urban clients. Although 80.0% of rural clients already had at least 2–3 children, 58.3% still desired more children (similarly for urban clients, 69.2% already had 2–3 children but 70.0% still desired more children) (Table 2). Urban clients were less likely than rural clients to have more than 3 children than rural clients (*P* <0.001). Forty-three percent, at urban centers, and 50.0%, at rural centers, reported having ever had an unwanted pregnancy; nearly all (93.8%) accepting the pregnancy (overall, only 3 participants reported successfully aborting).

**Table 1:** Characteristics of family planning and antenatal clinic clients participating in quantitative interviews

Characteristic	Urban (*N* = 120)	Rural (*N* = 60)	*P* ^a^
	*n* (%)	*n* (%)	
Age, median (IQR)	25 (23–30)	28 (24–32)	0.918
Catholic religious beliefs	34 (28.3%)	15 (25.0%)	0.064
Protestant religious beliefs	50 (41.7%)	40 (66.7%)	0.002
Other religious beliefs	36 (30.0%)	5 (8.3%)	0.001
Married/cohabiting	112 (93.3%)	56 (93.3%)	1.0
No or only primary-level education	57 (47.5%)	-	-
Secondary level education	43 (36.4%)	-	-
Tertiary level education	20 (16.7%)	-	-
Number of children			
0–1	37 (30.8%)	12 (20.0%)	0.124
2–3	60 (50.0%)	21 (35.0%)	0.057
≥ 4	23 (19.2%)	27 (45.0%)	<0.001
Desired at least one more child	84 (70.0%)	35 (58.3%)	0.119
Ever had an unwanted pregnancy	52 (43.3%)	30 (50.0%)	0.397
Accepted unwanted pregnancy	47 (90.4%)	28 (93.3%)	0.845

^a^Compares the urban clients to the rural clients

**Table 2:** Perceptions and practices of participants regarding contraceptive methods

Characteristic	Urban (*N* = 120)	Rural (*N* = 60)	*P*
	*n* (%)	*n* (%)	
Knowledge of methods			
Injectables	120 (100.0%)	59 (98.3%)	0.152
OC pills	120 (100.0%)	57 (95.0%)	0.014
Condoms	120 (100.0%)	52 (86.7%)	<0.001
Implants	117 (97.5%)	55 (91.7%)	0.082
IUD	103 (85.8%)	36 (60.0%)	0.001
Permanent methods (BTL and vasectomy)	86 (71.7%)	31 (51.7%)	0.043
Knowledge of whether methods were long-acting or short-acting.			
Correctly listed long-acting methods as such	85 (70.8%)	12 (20.0%)	<0.001
Correctly listed short-acting methods as such	89 (74.2%)	21 (35.0%)	<0.001
Knowledge of how methods are used			
Knew how at least one method is used	120 (100.0%)	60 (100.0%)	1.0
Knew how all listed methods are used	31 (25.8%)	5 (8.3%)	0.006
Belief in myths about contraceptives			
Agreed with “contraceptives cause cancer”	64 (53.3%)	34 (56.7%)	0.564
Agreed with “contraceptives cause birth defects”	65 (54.2%)	22 (36.7%)	0.017
Agreed with “contraceptives cause infertility”	59 (49.2%)	20 (33.3%)	0.034
Which type of method is more effective?			
LARC methods	75 (62.5%)	35 (58.3%)	0.543
Short-acting methods	26 (21.7%)	12 (20.0%)	0.775
Did not know	19 (15.8%)	13 (21.7%)	0.275
What do you think is the least effective method			
Withdrawal method	62 (51.7%)	14 (23.3%)	<0.001
Oral contraceptive pills	41 (34.2%)	22 (36.7%)	0.770
Condoms	13 (10.8%)	13 (21.7%)	0.054
What do you think is the most effective method			
Implants	34 (28.3%)	18 (30.0%)	0.870
Injectables	33 (27.5%)	18 (30.0%)	0.777
Sterilization (BTL/vasectomy)	31 (25.8%)	17 (28.3%)	0.770
Condoms	3 (2.5%)	2 (3.3%)	0.763
Oral contraceptive pills	3 (2.5%)	1 (1.7%)	0.709
Ever used a LARC method	23 (19.2%)	19 (31.7%)	0.062
Ever used a short-acting method	104 (86.7%)	47 (78.3%)	0.191
Ever used oral contraceptive pills	39 (32.5%)	14 (23.3%)	0.20
Ever used injectable contraceptive	86 (71.7%)	39 (65.0%)	0.450
Ever used other short-acting methods (condoms, spermicides, moon beads)	10 (8.3%)	4 (6.7%)	0.920
Ever used implant	18 (15.0%)	14 (23.3%)	0.550
Ever used IUD	5 (4.2%)	3 (5.0%)	0.958
Talked to partner prior to use of contraceptive method	105 (87.5%)	53 (88.3%)	0.885
Partner supportive of contraceptive use	92 (76.7%)	50 (83.3%)	0.356

#### Knowledge of contraceptive methods

Awareness of contraceptive methods in both urban and rural participants was high. Participants were less familiar with IUD and sterilization methods than other forms of contraception (Table 2). More urban participants than rural participants correctly identified both short-acting methods and long-acting methods (74.2% vs. 35.0% (*P* < 0.01) for short-acting and 70.8% vs 20.0% (*P* < 0.001) for long-acting methods). All participants correctly stated how at least one method is used, but very few, especially at rural centers were able to state how all the listed methods are used (25.8% at urban centers vs 8.3% at rural centers, *P* = 0.006) (Table 2).

#### Attitudes towards contraceptive methods

The majority of participants believed that LARC methods were more effective than short-acting methods. However, when it came to ranking individual methods, implants and injectables were ranked as the most effective methods by a similar number of participants. There was a slight disagreement regarding how participants from urban and rural centers ranked methods as least effective. While the largest number of urban participants (51.7%) ranked the withdrawal method as least effective, followed by OC pills (34.2%), a larger number of rural participants (36.7%) ranked OC pills as the least effective, followed by withdrawal (23.3%) (Table 2).

#### Practices regarding contraceptive methods

A hundred and one (56.1%) clients were currently using a contraceptive method and were attending family planning clinics; 79 (43.9%) were not currently using a contraceptive method and were attending antenatal care clinics for an ongoing pregnancy. Most current users had used their method for a short period; only 16.3% had used their method for over 5 years. Only one client had used a permanent method (tubal-ligation) (Table 3).

**Table 3:** Family planning practices among non-pregnant women who were currently using contraceptive methods

Type of method	Urban (*N*=)	Rural (*N*=)	*P*
	*n* (%)	*n* (%)	
Short-acting methods	51 (42.5%)	25 (41.7%)	0.947
LARC methods	12 (10.0%)	13 (21.7%)	0.433
Type of short-acting methods			
Injectable	38 (31.7%)	15 (25.0%)	0.631
Oral contraceptive pills	8 (6.7%)	6 (10.0%)	0.823
Others (condoms, spermicides, moon beads)	5 (4.2%)	4 (6.7%)	0.868
Type of LARC			
Implants	10 (8.3%)	11 (18.3%)	0.504
Intra-uterine device	2 (1.7%)	1 (1.7%)	1.0
Bilateral tubal ligation	0	1 (1.7%)	-

#### Use of LARC methods

The overall prevalence of ever-use of LARC was low at 23.3%, with a relatively higher prevalence (31.7%) among rural than urban clients (19.2%) (*P* = 0.062). Among the LARC methods, implant was more used than the IUD; use of these methods did not significantly vary by whether clients were rural or urban (Table 2).

#### Use of short-acting methods

The overall prevalence of ever-using short-acting methods was 83.9%. The injectable was the most common ever-use method, followed by oral contraceptive pills. The injectable was also the most common currently used method (Table 3). Among the current users, choice of methods did not significantly vary by whether clients were urban or rural (Table 3).

#### Reasons for choosing LARC methods

Among participants using LARC methods, the most common reasons for choosing a LARC method was longer protection, followed by better choice for child spacing, effectiveness, and wanting a method that does not require daily application (Table 3).

#### Reasons for not choosing long-acting methods among short-acting methods users

Among the participants using short-acting methods, the most common reasons for not using a LARC method included wanting a client-controlled method and intending to conceive in the near future. Urban clients were less likely to opt out of a long term method due to opposition from partners (*P* = 0.039); rural clients’ were more likely to indicate lack of awareness as the reason for not using long-acting methods (*P* = 0.04) (Table 4).

**Table 4:** Common reasons for choosing, not choosing, and switching methods among ever users of long- and short-term methods

Reasons suggested by LARC method users for choosing a long-term method	Urban (*N* = 22)	Rural (*N* = 19)	*P*
	*n* (%)	*n* (%)	
Longer protection	17 (77.3%)	18 (94.7%)	0.026
Better choice for child spacing	17 (77.3%)	16 (84.2%)	0.231
Better effectiveness	18 (81.8%)	14 (73.7%)	0.581
Needed method not requiring daily application	16 (72.7%)	12 (63.2%)	0.592
More comfort and less worries during use	15 (68.2%)	12 (63.2%)	0.407
Reason suggested by short-acting methods users for not choosing a long-acting method	Urban (*N* = 98)	Rural (*N* = 41)	*P*
	*n* (%)	*n* (%)	
Needed a method they can control themselves	74 (75.5%)	28 (68.3%)	0.385
Intended to conceive in near future	67 (68.4%)	25 (61.0%)	0.507
Did not know the long-term methods	31 (31.6%)	16 (39.0%)	0.040
Opposition from partners to long-term methods	18 (18.3%)	14 (34.1%)	0.039
Unavailability of long-term methods	14 (14.3%)	11 (26.8%)	0.079
Long-term methods are expensive	15 (15.5%)	5 (12.2%)	0.694
No trained staff to give method	10 (10.2%)	9 (22.0%)	0.057
Other reasons (e.g., side effects)	10 (10.2%)	2 (4.9%)	0.325
Reason suggested by short-acting methods users, as well as some of the long-acting methods users who had used short-acting methods in the past for choosing a short-term method	(*N* = 104)	(*N* = 47)	*P*
	*n* (%)	*n* (%)	
Ease of access	95 (84.8%)	41 (78.9%)	0.344
Method is cheap	91 (87.5%)	32 (68.1%)	0.010
Freedom to stop use without involving health provider	87 (83.7%)	28 (59.6%)	0.001
Privacy	80 (76.9%)	33 (70.2%)	0.918
Fewer side effects	71 (68.3%)	31 (66.0%)	0.367
Did not know about other methods	22 (21.2%)	15 (31.9%)	0.121
Can be used as a temporary or back up method	13 (11.9%)	16 (34.8%)	<0.001

#### Reasons for choosing short-acting methods

Among the same participants, the most common reasons for choosing short-acting methods were: accessibility; lower cost; privacy; and freedom to stop use without first seeing a health care worker. Compared to rural clients, urban clients were more likely to choose a short term method because of cost (*P* = 0.01) and because they could easily stop it without involving a provider (*P* = 0.001). (Table 4).

#### Factors associated with use of LARC methods

We assessed proportions using long-acting contraceptive methods by different characteristics among both rural and urban clients. In both the urban and rural setting, the proportion using LARC methods appeared to increase with age. Also, a larger proportion of those with more than 1 child had ever used LARC methods. Although urban clients seemed to suggest that they were less likely to opt out of using a LARC due to partner opposition, a larger proportion of urban clients with supportive partners had ever used LARC methods. Finally, among clients in the urban setting (where education information was collected), proportions using LARC methods increased with education levels (14% in those with primary or no education, 16% in those with secondary education, and 40% in those with tertiary education) (Table 5).

**Table 5:** The association between selected client characteristics and LARC ever-use among urban and rural clients (*n* = 180)

Client characteristic	Urban (*N* = 120)	*P* ^a^	Rural (*N* = 60)	*P*
	*n* (%)		*n* (%)	
Age (Years)				
17–24	7 (12.5%)	Ref.	3 (17.7%)	Ref.
25–29	6 (19.4%)	0.394	8 (36.4%)	0.206
30–49	10 (30.3%)	0.044	8 (38.1%)	0.176
Education status^b^				
None or primary	8 (14.0%)	Ref.	-	-
Secondary level	7 (16.3%)	0.784	-	-
Tertiary level	8 (40.0%)	0.014	-	-
Number of children				
0–1	5 (13.5%)	Ref.	2 (16.7%)	Ref.
2–3	10 (16.7%)	0.677	7 (33.3%)	0.310
≥ 4	8 (34.8%)	0.059	10 (37.0%)	0.216
Still desired children				
No	11 (30.1%)	Ref.	11 (44.0%)	Ref.
Yes	12 (14.3%)	0.042	8 (22.9%)	0.087
Partner supportive of contraceptive use				
No	3 (11.1%)	Ref.	4 (36.4%)	Ref.
Yes	20 (21.5%)	0.236	15 (30.6%)	0.711
Agreed with contraceptives cause cancer				
No	8 (16.7%)	Ref.	5 (23.8%)	Ref.
Yes	14 (21.9%)	0.493	12 (35.3%)	0.373
Agreed with contraceptives cause birth defects				
No	11 (21.2%)	Ref.	13 (34.2%)	Ref.
Yes	11 (16.9%)	0.561	6 (27.3%)	0.578
Agreed with contraceptives cause infertility				
No	11 (18.6%)	Ref.	14 (35.0%)	Ref.
Yes	11 (18.6%)	1.0	5 (25.0%)	0.434

^a^Compares the proportion in the other categories to the reference category among the urban clients. The same comparison was performed among the rural clients

^b^Data was collected for only the urban clients (*N* = 120)

### Qualitative data

#### General perceptions of modern contraceptive methods among focus group discussion participants

From the focus group discussions, the majority of participants identified short-acting and LARC methods appropriately. Overall, we did not observe any knowledge differences between focus group discussion participants based on whether they were urban or rural. However, clients tended to be knowledgeable about a method if they had ever used it. Also, clients using LARC methods (implants and IUDs) were generally more knowledgeable about contraceptive methods than those using short-acting methods (pills, injectables or/and condoms). In particular, those using short-acting contraceptives lacked specific information on how LARC work. For example, one focus group discussion participant among short-term users said: *“The coil (IUD) works in the uterus by blocking the tubes such that when the eggs come from the tubes they get pricked by the coil and hence they break” (Client using injectables at Mbarara Municipal Council health center IV-Urban health center).*

Focus group discussion participants had both negative and positive perceptions of different contraceptive methods. On the negative side, many of those using short-acting methods had at least one concern, usually more myth than reality, about LARC methods. For example, they seemed to worry a lot about possible long-term side effects. One client said *“I fear the long-term side effects and cancer since one has to stay with the method for a long time”. (Young female client, focus group discussion at Mbarara Municipal Council IV- Urban health center)*. Some also worried that LARC methods may affect physical activities and fertility after use. One said: *“Even the injections cause loss of periods while you are using them; I am afraid that if I use a long-term method like the Implant, I might never bleed again” (Young client at Kinoni health center IV-Rural health center)* (in this setting, the term “bleeding” is often used to refer to fertility).

On the positive side, some clients disagreed with the worries on physical activity, with one saying: *“Before I started using the Implants, some women used to say you cannot lift something heavy while using the implant, but I did not find any problem because I can even go to the gardens and do my farming as usual without any problem” (Client using an Implant at Kinoni health center IV-Rural health center).* Another client expressed disappointment about others who blame all their problems on contraceptives: *“Some women are preoccupied with the symptoms told to them during the family planning training before starting to use the methods, hence they tend to blame everything that happens to them afterwards on family planning which may not always be the case” (Young female client using an Implant, focus group discussion at Mbarara Municipal Council health center IV- Urban health center).*

#### Factors influencing method selection

From both the focus group discussions (with clients) and in-depth interviews (with health workers), several themes relevant to method-selection emerged as follows.

#### Level of knowledge

In general, those more knowledgeable about contraceptives were more likely to harbor positive attitudes towards long-term methods and hence were more likely to use them. Health worker interviews supported this. One said: *“Many women have a low level of education. The long-term methods are used by educated people who even have the capability to read about the methods from the internet before they proceed to use the method” (Female Mid-wife, 55 year old, MRRH-Urban health center).* Another health worker said: *“Being an urban area some clients we get are elite, they come and request for the long-term methods” (Female Senior Nursing Officer-Midwife, Kakoba health center III-Urban health center)*.

#### Myths and fears

There were several myths and fears among the clients, which seemed to influence method selection. For example, one client said: *“I hear some people saying when you use injectables, the uterus loosens and you start feeling it all over the abdomen, although for me I did not experience that”. (Client at Kinoni health center IV-Rural health center).* Another said of a related myth: *“Some people have their own thinking, they tell you for example if I only have a few eggs and I use a long-term method, by the time I want to conceive the eggs may be over and I fail to conceive” (Client at Mbarara Municipal Council health center IV-Urban health center).* Cancer appeared to be one of the major fears. Of this, one client said thus: *“A woman in my village who was using the implants suffered from cancer of the cervix, I cannot risk using the implants” (Client at Kinoni health center IV-Rural health center).* A health worker at a rural facility appeared to corroborate this fear of cancer: *“Women say that the IUDs cause cancer, especially arising from what they have heard from their friends” (Female, Midwife, Kinoni health center IV-Rural health center).* At an urban facility, another health worker said: *“The mothers have got their myths and fears; they fear they may fail to conceive later. So those who go for long-term methods are sure they are through with delivering and therefore do not mind a lot about their subsequent fertility”. (Female Senior Nursing officer-Midwife, Kakoba health center III-Urban health center)*.

#### Past experiences

A number of clients based the effectiveness of a given method on their past experience with side effects or failure. In turn, this appeared to influence whether or not they selected a method. For example, one said: *“While I was on Injectaplan, I was disappointed because I got pregnant when I thought I was using a method of contraception” (Young Female client, focus group discussion at Mbarara Municipal Council health center IV - Urban health center).* Health workers also talked about past experiences influencing knowledge and choice of method. One health worker said: *“Most women have used the injectable and are hence more knowledgeable and more confident about using them from their own experiences. Also, because many other women are using the same method, the clients can benefit from the experiences of their friends” (Female Mid-wife, 55 year old, MRRH-Urban health center)*.

#### Availability of method

Both clients and health workers mentioned the wide availability of short-acting contraceptive methods and the unavailability of LARC methods as important in their ability to use or not to use a method. In particular, LARC methods were considered unavailable in the rural health centers, especially by clients. One said: *“At the Health Centre the long-term methods are not available. We usually wait for an announcement by Blue Star (a local program that provides long-acting contraceptive methods) that the services are being brought, and that’s when we come to the health center” (Young Female client, focus group discussion at Kinoni health center IV-Rural health center).* Another respondent agreed: *“The methods offered at this Health Centre are pills, condoms, and injectables, which are adequately supplied because we have never come here and not found them” (Young female client, focus group discussion at Kinoni health center IV- Rural health center).*

Also, especially at the rural health centers, the inadequacy of trained health workers to implement methods or the facilities required was commonly mentioned. For example, one health worker said: *“The nurse who had been trained to do the insertion and removal of IUDs and Norplant at this Health Centre was transferred leaving the facility with the equipment which have been here unused for up to 5 years now*” *(Female middle-aged Senior Nursing Officer, Bwizibwera health center IV-Rural health center)*. LARC methods appeared to be more available at urban health centers. For example, a health worker from an urban facility said: *“We have the short-term methods, so we have the pills; emergency pills, combined oral contraceptives, condoms, those are usually available, but mothers prefer the injectables. Long-term methods are also available.” (Female, middle-aged Midwife, Mbarara Municipal Council health center IV- Urban health center).* However, the inadequacy of trained human resources and facilities required to implement long-term methods seemed to also be a problem at the urban facilities. One health worker said: *“We also have IUDs but we have not been inserting them because our sterilizer had a fault” (Female middle-aged Senior Nursing Officer, Mbarara Municipal Council health center IV-Urban health enter).* At another facility, a health worker said: *“Instruments are also not enough. We only have 3 instrument sets for IUD insertion. Speculums are lacking and the few we have are old” (Female Mid-wife, MRRH-Urban health center).*

Especially in the rural setting, perceptions regarding availability of methods varied between clients and health workers. From the perspective of clients, the LARC methods were largely unavailable (they therefore had to “*wait for a radio announcement to know when the methods would become available before visiting the health centers to ask for such methods*”). On the other hand, according to the health workers, the methods were mostly available; the problem was lack of either trained personnel or facilities required to implement the methods.

#### Side effects

Side effects seemed to influence stoppage rather than method selection. The side effects commonly mentioned were excessive bleeding and lack of periods. Said one client of this: “*There are side effects from the different methods, for example I bled so much when I was using an implant; this forced me to leave the method” (Young Female client, focus group discussion at Kinoni health center IV- Rural health center).*

#### Number of children

Number of children also influenced method selection. Said one client of this: *“Now for me I have only one child, I need to use methods that are not taking a long time. I cannot use the IUD which stays for 10 years” (Young Female client, focus group discussion at MMC health center IV-Urban health center).* This client was evidently unaware that the IUD could be removed before 10 years if she so desired. A health worker also noted that parity of the women influenced their choice of method: *“Many mothers over 35 years and those that have 4 to 5 children opt for the long term methods including the permanent ones” (Female Senior Nursing officer-Midwife, Kakoba health center III-Urban health center).*

#### Privacy, cost, and partners

Additional, but less prominently featured themes were: privacy, cost, and partners. For example, commenting on why some clients may not use IUDs, a health worker said: *“Most clients do not like IUDs because they do not like someone tampering with their private parts” (Female middle-aged Senior Nursing Officer, Mbarara Municipal Council health center IV- Urban health center).* Said another health worker: *“Most people use Depo-Provera because it is readily available and it is also more private. Most mothers tell us that they are hiding from their spouses, since they do not want them to use any family planning methods” (Female middle-aged Senior Nursing Officer, Mbarara Municipal Council health center IV-Urban health center)*. This was corroborated by a client who said: *“We use the injectable illegally, because our husbands do not want us to use family planning, we therefore cannot use long-term methods” (A 21 year old female client using Depo-Provera injectable at Kinoni health center IV-Rural health center).* A health worker seemed to agree*: “There is poor male involvement in family planning, hence women prefer injectables which they can use when their husbands are not aware” (Female Senior Nursing officer-Midwife, Kakoba health center III-Urban health center).*

Finally, some focus group discussion participants raised cost as an important factor. One said: *“Most of the women do not have the money to pay for implants and IUDs which are expensive, so they prefer to use the pills and injectables that are always available at the Health Centre and are cheap, costing about 1,000 or 2,000 Uganda shillings” (Young female client, focus group discussion at Kinoni health center IV-Rural health center).*

## Discussion

Increased use of LARC methods is a cost effective approach to preventing unwanted pregnancy and reducing maternal mortality in resource-limited settings [8, 12]. Whereas there is need to adequately provide all contraceptive methods, we expect that LARC methods may have a better chance at averting the unmet need for contraception in resource-limited settings [13]. This is because, compared to short-acting methods, LARC methods are more efficacious, provide better child spacing, are more cost-effective, and their effectiveness tends to be independent of user characteristics [6, 14].

Among reproductive-age women in Uganda, we assessed the prevalence of using LARC and short-acting contraceptive methods and evaluated factors influencing selection of LARC methods versus short-acting contraceptive methods. The prevalence of ever use for LARC methods was 23.3%, an improvement on estimates from previous studies (3.2%) [3], but which still suggests limited use of LARC methods. LARC methods were also more used in the rural setting (31.7%) than in the urban setting (19.2%) despite the methods being relatively more accessible in the latter setting.

In this study, we particularly aimed to assess whether the factors influencing method selection were predominantly structural, for example, unavailability of methods or lack of trained personnel to implement them [8], or method-related, for example, ease of application, side effects, and cost [15], or related to the personal characteristics of clients, for example, resistance from partners, lack of knowledge, or holding certain beliefs, right or wrong, about contraceptive methods [16].

From the quantitative results, method characteristics appeared to stand out. For example, commonly mentioned reasons for choosing or not choosing a method revolved around method characteristics such as, client control, privacy, freedom to stop use without a health worker, ease of access, cost, and side effects. Personal client characteristics such as number of children were also relatively important. In contrast, from the qualitative results, structural factors, that is, availability of both the methods and trained personnel to implement the methods appeared to be more important.

Overall though, method characteristics seemed to be most important. In both rural and urban settings, reasons for choosing or not choosing any method were predominantly method-related. Furthermore, while rural clients had suggested that they were not using LARC methods because of unavailability, at the urban clinics, where the LARC methods were relatively available, clients still opted to use short-acting methods. Among the method characteristics, client-control and ease of application were most desired, which is consistent with some prior studies. In a qualitative study assessing knowledge and perceptions regarding long-acting and permanent contraceptive methods in urban Ethiopia, women preferred methods that did not require any procedure [17].

These findings suggest that addressing structural barriers hindering access to contraceptive services may increase uptake of LARC methods [8]. However, an alternative or complementary approach may be to incorporate preferred characteristics into newer or existing LARC methods. Some existing methods such as contraceptive patches and vaginal rings provide a degree of long-term protection and are relatively client-controlled [18], but these methods are not yet being marketed in many resource-limited settings. A disadvantage of such an approach is that the effectiveness of LARC methods is premised on their being user-independent. It therefore is unclear whether LARC methods that are more client-controlled would retain their efficacy, especially in a resource-limited setting.

Certainly, the role of structural factors cannot be overlooked [19]. At both rural and urban clinics, there was at least one problem with availability of LARC methods. In the rural setting, according to clients, LARC methods were mostly unavailable. According to the health workers, the methods were mostly available, but there were no trained personnel to implement them or there was one or other problem with the additional facilities required. This varying interpretation of structural barriers by clients and health workers at rural clinics may reflect a communication gap [20]. Also, in the urban setting, where LARC methods were relatively more available, there was either unavailability or inadequacy of facilities to implement them, suggesting a gap in the targeting of supplies [9]. Ideally, availability of LARC methods should correlate with availability of trained personnel and facilities to implement such methods [8].

Structural factors may also explain why particular method characteristics were preferred. For example, the absence of trained health professionals and equipment could explain why clients prefer methods that they can stop without involving a health worker. Women might fear to insert a coil if they feel that there will be no one to insert it properly or if they are worried that removing it might be difficult [17]. The lack of knowledge about LARC methods by some clients may also reflect inadequacy of training for health workers on how to educate clients appropriately, or lack of time to perform appropriate client sensitization due to health workers being few and busy [21]. In the urban centers, clients seemed to rank method effectiveness more correctly than in the rural centers (e.g., many rural clients ranked withdrawal as more effective than oral contraceptive pills), which may reflect variation in knowledge across the two settings.

Addressing such structural barriers could improve use of LARC. However, more studies are required to identify optimal and cost-effective approaches to doing this. For example, the numbers of trained health providers at these facilities would need to increase [19]; how to best do this remains unclear [22, 23]. Furthermore, it is important to increase and ensure a steady and reliable supply of LARC methods and the other material facilities required to implement the methods [24]. The targeting of supplies could be improved, by, for example, supplying LARC methods to only those centers where there are trained health workers and other facilities required to implement the methods.

The personal characteristics of clients were also important in method selection. In the urban setting where education data were available, increasing education levels were associated with increased use of LARC methods. In both urban and rural settings, some women opted out of using LARC methods due to flat-out wrong beliefs. These findings suggest that the currently available LARC methods may not be accepted in part because of inadequate knowledge among reproductive age women. Sensitization of such women could therefore help. However, it also appears that increasing levels of education may improve usage of contraceptives and reduce risk of unwanted pregnancies. Notably, increasing education among women could mean that such women can use the short-acting methods even more correctly allowing such methods to be more effective. Education therefore is likely to increase the effective options available to women and facilitate increased client choice [24].

Whereas our quantitative data suggested that partners were supportive of contraception, the qualitative data appeared to go against this finding. In the qualitative data, there was a desire among women to hide their use of contraceptives from their partners. This is consistent with what has been observed in previous studies. In a Ugandan study, women believed that their partners should actually take part in the decision to use a method of contraception [16]. However, whether partners actually did play a positive role in these decisions was not clarified. In Cambodian study, women who believed that their husbands had a positive attitude towards contraception were more likely to use a method than those who did not have a similar belief about their husbands. In the same study women whose husbands made the final decision about contraception were less likely than others to use a method [25].

Another study in Zambia found that in circumstances where male partners voice different preferences and attitudes toward family planning, women may not use modern contraceptives or they may secretly obtain the methods or prefer to use concealable methods of contraception. In that study women were given vouchers to obtain frequently out of stock contraceptives (implants and injectables). Women were more likely to return for the different methods if they had received the vouchers without their husbands compared to if they had received the vouchers as a couple [26].

From these previous studies and our own data, it is evident that partner support is inadequate in this setting, and this could be because the partners, just like the women, lack knowledge about the different contraceptive methods. Although this issue should be the subject of future studies, sensitization of both women and their partners may be a good place to start.

Our findings have some limitations. Sample sizes for quantitative analyses were small. Also, a substantial proportion of respondents were seeking antenatal care services. Our sample may therefore have been enriched with clients more likely to use short-acting methods. However, our estimate of LARC methods use was higher than that found in previous studies; our findings also showed a similar pattern as that observed in population-based studies, i.e., more women using short-acting methods [3]. Additionally, education information was not collected from participants in the rural setting making it difficult for us to assess the role of education level in method selection. The women involved in our study were served at public facilities; we intended to collect data from government health centres where antenatal care and family planning services are provided free of charge. At these centers, services were provided from around 8 am to early afternoon (usually 2 pm). In contrast, women who seek family planning services from private health facilities in Uganda might be able to obtain the services at whatever time they choose. Since we did not collect the data of such women, our results may not be easily generalizable to populations of women who seek family planning services from private health facilities in Uganda. A strength of our study is that we assessed clients in multiple locations and used multiple methods of data collection. This allowed a richer understanding of the constructs under investigation and gave perspective to some of our observations.

## Conclusions

Among reproductive-age women in a resource-limited setting, the prevalence of LARC use remains low. Method selection appeared to be predominantly influenced by method-related characteristics, although personal characteristics and structural factors were also important. The method-characteristics that appeared to be most desired were client-control and ease of application. However, we cannot rule out that this preference of specific method characteristics is driven by structural barriers such as the lack of facilities to appropriately administer LARC methods. Future studies should assess best approaches to addressing some of such structural barriers hindering access to LARC methods. In particular, provision of LARC methods should be tied to availability of resources to implement the methods. Better client sensitization and counselling, as well as increased education among women, may also improve uptake of LARC methods in this setting.

## Abbreviations

ANC: Antenatal care
FGD: Focus group discussion
FP: Family planning
HC: Health center
IUD: Intrauterine device
LARC: Long-acting reversible contraceptive
MMC: Mbarara Municipal Council
MRRH: Mbarara Regional Referral Hospital
